# Nox2 Regulates Platelet Activation and NET Formation in the Lung

**DOI:** 10.3389/fimmu.2019.01472

**Published:** 2019-07-05

**Authors:** Jessica S. Hook, Mou Cao, Renee M. Potera, Nesreen Z. Alsmadi, David W. Schmidtke, Jessica G. Moreland

**Affiliations:** ^1^Department of Pediatrics, UT Southwestern Medical Center, Dallas, TX, United States; ^2^Department of Bioengineering, The University of Texas at Dallas, Richardson, TX, United States; ^3^Department of Microbiology, UT Southwestern Medical Center, Dallas, TX, United States

**Keywords:** neutrophil, NET formation, platelet, Nox2, lung injury

## Abstract

The mortality rate of patients with critical illness has decreased significantly over the past two decades, but the rate of decline has slowed recently, with organ dysfunction as a major driver of morbidity and mortality. Among patients with the systemic inflammatory response syndrome (SIRS), acute lung injury is a common component with serious morbidity. Previous studies in our laboratory using a murine model of SIRS demonstrated a key role for NADPH oxidase 2 (Nox2)-derived reactive oxygen species in the resolution of inflammation. Nox2-deficient (gp91^phox−/y^) mice develop profound lung injury secondary to SIRS and fail to resolve inflammation. Alveolar macrophages from gp91^phox−/y^ mice express greater levels of chemotactic and pro-inflammatory factors at baseline providing evidence that Nox2 in alveolar macrophages is critical for homeostasis. Based on the lung pathology with increased thrombosis in gp91^phox−/y^ mice, and the known role of platelets in the inflammatory process, we hypothesized that Nox2 represses platelet activation. In the mouse model, we found that platelet-derived chemokine (C-X-C motif) ligand 4 (CXCL4) and CXCL7 were increased in the bronchoalveolar fluid of gp91^phox−/y^ mice at baseline and 24 h post intraperitoneal zymosan-induced SIRS consistent with platelet activation. Activated platelets interact with leukocytes via P-selectin glycoprotein ligand 1 (PSGL-1). Within 2 h of SIRS induction, alveolar neutrophil PSGL-1 expression was higher in gp91^phox−/y^ mice. Platelet-neutrophil interactions were decreased in the peripheral blood of gp91^phox−/y^ mice consistent with movement of activated platelets to the lung of mice lacking Nox2. Based on the severe lung pathology and the role of platelets in the formation of neutrophil extracellular traps (NETs), we evaluated NET production. In contrast to previous studies demonstrating Nox2-dependent NET formation, staining of lung sections from mice 24 h post zymosan injection revealed a large number of citrullinated histone 3 (H3CIT) and myeloperoxidase positive cells consistent with NET formation in gp91^phox−/y^ mice that was virtually absent in WT mice. In addition, H3CIT protein expression and PAD4 activity were higher in the lung of gp91^phox−/y^ mice post SIRS induction. These results suggest that Nox2 plays a critical role in maintaining homeostasis by regulating platelet activation and NET formation in the lung.

## Introduction

Despite numerous technological advances in intensive care in recent years, mortality from sepsis and the systemic inflammatory response syndrome (SIRS) remains static. The World Health Organization made sepsis a global health priority in 2017 based on the burden of disease with an incidence of 750,000 cases/year in the USA. Although the mortality rate has decreased substantially, more than 25–30% of patients with sepsis will die from complications of multi-organ injury (1). Among many organ systems at risk during SIRS, acute lung injury and the resultant acute respiratory distress syndrome is a common cause of severe morbidity. Mechanical ventilation and treatment of the inciting stimulus are the most common therapies, but there are no current effective therapies targeting underlying mechanisms (2, 3). Acute respiratory distress syndrome is characterized by acute and diffuse inflammation in the lung with rapid neutrophil infiltration and pulmonary edema. The contribution of neutrophils to the lung injury process is well-recognized with exuberant cell activation and the release of both reactive oxygen species (ROS) and proteolytic granule contents (4).

Early studies in our laboratory suggested a novel anti-inflammatory role for the neutrophil NADPH oxidase 2 (Nox2) in the pathogenesis of acute respiratory distress syndrome as demonstrated by the severe lung pathology that developed in mice lacking Nox2 after a systemic inflammatory stimulus (5). Wild type (WT) and Nox2 deficient (gp91^phox−/y^) mice develop SIRS in response to intraperitoneal injection of zymosan, but only gp91^phox−/y^ mice develop acute and severe lung pathology with inflammation, disseminated thrombosis, and hemorrhage. Margination of neutrophils is seen in the lungs of both WT and gp91^phox−/y^ mice, but only the mice lacking Nox2 have infiltration of neutrophils in the alveolar space (6). A decrease in circulating platelet concentration is seen within 2 h in gp91^phox−/y^ mice with concomitant generation of microthrombi in the lung and hemorrhagic bronchoalveolar (BAL) fluid (7). Considered in combination, these studies suggested that Nox2 derived ROS serve to repress the inflammatory response in the lung.

The notion that Nox2 deficiency could lead to a pro-inflammatory state is certainly not novel. Patients with chronic granulomatous disease lacking Nox2 function display a number of hyperinflammatory phenotypes; *in vitro* analysis of PMN demonstrates upregulation of CD11b and phosphorylation of p38 MAPK at baseline (8). *In vivo*, patients with chronic granulomatous disease experience frequent infections and chronic inflammation suggesting a role for Nox2 in limiting and resolving inflammation. Chronic granulomatous disease is characterized by the presence of granulomas, masses of immune cells at the sites of inflammation and infection. Granulomas have been described in numerous tissues and organs including the lung, bladder, eyes, and gastrointestinal tract (9). In broad terms, neutrophil recruitment to tissues and organs, whether in response to sterile inflammation as in a granuloma or in response to infection, is governed by a coordinated interplay of chemoattractants and adhesion molecules. Ideally, neutrophils are rapidly recruited to a site of infection and then abruptly called off with resolution of the inciting stimulus.

Our laboratory demonstrated upregulation of alveolar macrophage chemokine production in Nox2-deficient mice (7), but these tissue macrophages are not the only source of chemokine production. CXC chemokine ligand 4 (CXCL4) and CXCL7, chemokines released by activated platelets, are strong neutrophil chemoattractants (10). P-selectin glycoprotein 1 (PSGL-1) is expressed on leukocytes and plays an important role in cell adhesion and migration through the endothelium. PSGL-1 binds to the cell adhesion molecule, P-selectin, expressed on activated endothelial cells and activated platelets (11). CXCL4, CXCL7, and P-selectin (12, 13) are stored in the alpha granules of platelets and released or upregulated to the platelet surface upon activation (11, 14). In addition, to functioning as a chemokine, CXCL4 has been demonstrated to induce the formation of neutrophil extracellular traps or NETs (15, 16).

NETs were first described 14 years ago as a novel mechanism of cell death that served an antimicrobial function (17). A much broader role for NETs is now recognized including involvement in the pathophysiology of chronic inflammatory processes ranging from rheumatoid arthritis to gout, suggesting NET formation may contribute to tissue damage (18, 19). NET formation has been observed *in vivo* and in response to numerous stimuli *in vitro*, and the activation pathways leading to NET formation vary by stimulus (18, 20). Although it was initially perceived that NADPH oxidase generated ROS were required for NET formation, it is now recognized that ROS-dependence is stimulus dependent (21). Histone H3 citrullination (H3CIT) by active peptidylarginine deiminase 4 (PAD4) is required for NET formation to some stimuli but not all (18). Among the various stimuli, activated platelets can elicit NET formation in the absence of ROS (18). In addition, there is recent evidence that NETs contribute to the pathogenesis of lung injury and that NET formation in the lung is mediated by platelet-neutrophil interactions (22).

In this study, we extend our previous findings of a protective role for Nox2 in the development of lung injury and provide a better understanding of the underlying mechanisms whereby Nox2 limits and resolves inflammation in the lung. We found that Nox2 limits platelet activation and the upregulation of adhesion molecules. In our model, NET formation occurred only in the lungs of Nox2-deficient mice, suggesting that Nox2 specifically represses NET formation under homeostatic conditions. In the absence of Nox2, multiple cell types lose this homeostatic repression; platelet and macrophages release neutrophil chemoattractants and there is rapid infiltration of activated neutrophils in the setting of activated platelets. These concurrent events allow the formation of NETs that lead to extensive tissue damage. A better understanding of the role of Nox2-derived ROS in limiting and resolving inflammation is critical to developing targeted therapies for the treatment of lung injury secondary to systemic inflammation.

## Materials and Methods

### Materials

Zymosan A from Sigma-Aldrich (St. Louis, MO, USA) was prepared as previously described (7). Ly6G (clone 1A8), CD41 (clone MWReg30), CD45 (clone 30-F11), PSGL-1 (clone 4RA10), and ICAM-1 (clone HA58) antibodies were purchased from BD Biosciences (San Jose, CA, USA). Collagenase D, DNase I, and tetramethylbenzidine were purchased from Sigma-Aldrich. CXCL4 (clone 140910), CXCL7 (clone 159703), and myeloperoxidase (clone 392105) antibodies, streptavidin HRP, and recombinant mouse P-selectin (CD62P Fc chimera recombinant protein) were purchased from R&D Systems (Minneapolis, MN, USA). PAD4 (clone EPR20706) and H3CIT (ab5103) antibodies were purchased from Abcam (Cambridge, MA, USA). Ly6G-biotin antibody, MicroBeads, and columns were purchased from Miltenyi Biotec (San Diego, CA, USA). Paraformaldehyde was purchased from Electron Microscopy Sciences (Hatfield, PA, USA). All other reagents and supplies were purchased from Fisher (Waltham, MA, USA).

### Animals

All studies were approved and conducted under the oversight of the Institutional Animal Care and Use Committee at the University of Texas Southwestern Medical Center. C57BL/6J (WT) and Nox2-deficient (B6.129S-*Cybbtm1Din*/J) mice (gp91^phox−/y^) were obtained from Jackson Laboratory (Bar Harbor, ME, USA) and kept in a barrier facility with free access to standard rodent chow and water. *CYBB* encodes gp91^phox^ and is present on the X chromosome. CGD resulting from a mutation in CYBB is inherited in an X-linked recessive pattern; therefore, only male mice were used. Mice were age matched for all experiments.

### Zymosan-Induced Generalized Inflammation

Sterile systemic inflammation was induced using the zymosan-induced generalized inflammation model, in which mice received an intraperitoneal injection of zymosan (0.7 mg/g) as previously described (5). Mice were sacrificed at 2 and 24 h post injection.

### Evaluation of Lung Injury

BAL was performed as previously described (7). Following BAL, the pulmonary vasculature was perfused through the right ventricle with 5 ml of phosphate buffered saline (PBS). Lungs were removed, cut into small pieces with scissors, and subjected to digestion in digestion buffer (1 mg/ml collagenase D and 0.1 mg/ml DNase I in PBS) for 30 min at 37°C with continuous shaking. Lungs were mechanically digested using a Miltenyi gentleMACS™ Dissociator from Miltenyi Biotec (San Diego, CA, USA) according to the manufacturer's directions. Homogenized lungs were passed through a 70 μm nylon filter, and the lung homogenate was centrifuged at 500 g for 5 min at 4°C. The resultant supernatant was collected and stored at −80°C. Miltenyi MicroBeads were used to isolate neutrophils from the digested lung according to manufacturer's directions.

### ELISA for CXCL4 and CXCL7

96-well NUNC Maxisorp microplates were coated with antibodies against CXCL4 or CXCL7 in carbonate buffer (100 mM NaHCO_3_, 34 mM Na_2_CO_3_, pH 9.6) overnight at room temperature. Wells were blocked with PBS containing 1% BSA and 5% sucrose for 1 h. Standards and samples were diluted in assay diluent (PBS with 0.1% BSA), loaded into duplicate wells, and incubated for 2 h at room temperature. Biotinylated antibody against the target was added and allowed to incubate for 2 h followed by streptavidin-HRP for 20 min. Tetramethylbenzidine was added to the wells for 10–30 min for color development and 0.5 M H_2_SO_4_ was added to stop the reaction. All incubations were performed at room temperature and wells were rinsed three times with wash buffer (PBS with 0.05% Tween 20) between each step. Absorbance at 450 nm was measured on a Clariostar Omega from BMG Labtech (Cary, NC, USA).

### Flow Cytometry

Neutrophil-platelet aggregates—Antibodies were added to whole blood and incubated on ice for 1 h. FACS lysis buffer from BD Biosciences (San Jose, CA, USA) was added and cells were mixed on ice for 10 min. Cells were then washed with 3 ml of cold PBS. Secondary antibodies were added where appropriate and incubated for another 30 min on ice. Cells were washed and resuspended in cold PBS and taken immediately to the flow facility for analysis. Cell surface markers—Isolated cells were fixed with 4% paraformaldehyde for 30 min on ice and blocked with PBS containing 4% normal goat serum and 2% non-fat dry milk on ice for 20 min. Cells were incubated with primary antibodies on ice for 1 h followed by washing and a 30 min incubation on ice with secondary antibody as needed. All samples were washed and resuspended in PBS prior to flow analysis. All data acquisition was performed on a BD FACSCalibur from BD Biosciences (San Jose, CA, USA) in the Flow Cytometry Facility at the University of Texas Southwestern Medical Center with ≥10,000 events collected per analysis. Data were analyzed using FlowJo Software, version 10.0.08 from Treestar (Ashland, OR, USA). Ly6G was used to identify neutrophils where appropriate.

### Microfluidic Flow Chamber Assays

Microfluidic flow chamber assays were performed to measure differences in the formation of neutrophil-platelet aggregates. For these experiments, the microfluidic flow chambers consisted of polydimethylsiloxane stamps that were permanently sealed to a glass coverslip by pretreatment with an air plasma (23). The microfluidic channels had a cross-sectional dimension of 300 μm wide by 50 μm tall and were fabricated by standard photolithography procedures described previously (23, 24). To measure the frequency of neutrophil-platelet aggregates, the microfluidic chambers were initially filled with Hanks' balanced salt solution and then coated with recombinant mouse P-selectin by perfusing a 0.2 μg/ml P-selectin solution through the channel at 1,600 s^−1^ for 15 min (25). The microfluidic channels were then blocked by filling the channels with a solution of 0.5% human serum albumin in Hanks' balanced salt solution and incubating for 30 min at room temperature before placing the microfluidic device on a Zeiss AxioVert A1 microscope equipped with a 63 × Plan-Apochromat (NA 1.4) objective and a Hamamatsu ORCA-Flash 4.0 scientific CMOS camera. Whole mouse blood anti-coagulated with 10 units/mL of heparin was then perfused through the channels at a shear rate of 100 s^−1^ and the rolling of leukocytes on the P-selectin coated surface was visualized by differential interference contrast microscopy and recorded at 30 frames per second. For each video recording (~5 s) we quantified the number of rolling leukocytes with and without adherent platelets. For those leukocytes with platelets adhered, we counted the total number of platelets.

### Confocal Microscopy

Mouse lungs were harvested 24 h post SIRS induction following BAL and circulatory perfusion as described above. The lung samples were rinsed with PBS immediately and fixed in 10% neutral buffered formalin overnight at 4°C. The fixed lungs were embedded in paraffin. Paraffin blocks were sectioned in 5-μm slices and deparaffinized followed by antigen retrieval with antigen unmasking solution from Vector Laboratories (Burlingame, CA, USA). The sections were blocked with 3% bovine serum albumin and incubated with primary antibodies at 4°C overnight. Sections were washed and incubated in appropriate secondary antibody for 2 h at room temperature followed by mounting. Images were acquired using a Zeiss LSM880 confocal microscope with Plan-Apochromat objectives at room temperature with Zeiss Immersol 518 F halogen free/fluorescence free imaging medium and processed using ZEN software from Carl Zeiss Microscopy (Thornwood, NY, USA). A 10 ×/0.8 air objective was used to acquire images for quantification of H3CIT staining. 10 fields at 10 × magnification per section were evaluated and H3CIT positive cells were counted and expressed as total number per field. A 40 ×/1.4 oil objective was used for the representative H3CIT images (**Figures 6B–E**), and a 63 ×/1.4 oil DIC objective was used for the representative PAD4 images (**Figures 8A,B**).

### Immunoblotting

Lung tissue was homogenized in protein lysis buffer (20 mM imidazole, 2 mM EGTA, 100 mM NaCl, 1% Triton X-100, 1 mM PMSF, 2% leupeptin/pepstatin A) using a Polytron Immersion Disperser from Kinematica (Bohemia, NY, USA) on medium to high speed for 20 s on ice. Homogenized samples were spun at 15,000 g for 10 min at 4°C to remove particulates. The protein lysate was collected, mixed with sample buffer and heated at 70°C for 10 min to denature proteins. TGX gels from Biorad (Hercules, CA, USA) were used to resolve proteins by SDS-PAGE followed by transfer to a nitrocellulose membrane. Membranes were blocked with 3% bovine serum albumin in tris-buffered saline containing 0.1% Tween 20 for 1 h at room temperature followed by overnight incubation in primary antibody at 4°C. Secondary antibodies were conjugated to horse radish peroxidase and detected by chemiluminescence using a ChemiDoc XRS Imager from Biorad. Bands were analyzed using Image Lab software from Biorad.

### Peptidylarginine Deiminase 4 Activity

Lung digest supernatant was harvested as described above. The PAD4 Inhibitor Screening Assay Kit (AMC) from Cayman Chemical (Ann Arbor, MI, USA) was used to assess PAD4 activity according to the manufacturer's instructions. Briefly, a fluorescent substrate consisting of an arginine, a carboxybenzyl group, and a fluorophore was incubated with the sample. In the absence of active PAD4, the substrate is unaltered and the fluorophore is released upon addition of the developer. In the presence of active PAD4, the substrate is citrullinated preventing the release of the fluorophore upon addition of the developer. Fluorescence was measured in a Clariostar Omega from BMG Labtech (Cary, NC, USA) with excitation set to 360 ± 15 nm and emission set to 450 ± 20 nm. Fluorescent signal is inversely proportional to PAD4 activity. Sample buffer alone was set to 0% activity and recombinant PAD4 enzyme was set to 100% activity. Sample activity was compared to the activity of the recombinant enzyme. Cl-amidine, an irreversible and cell-permeable pan PAD inhibitor, was used at a final concentration of 100 μM per the manufacturer's instructions. Cl-amidine potency is unstable and loses activity very quickly over time (21). For this reason, we used the inhibitor to validate specificity of the assay and not in a function blocking capacity.

### Statistics

All data are presented as mean ± SEM. Statistical analysis was performed using GraphPad Prism 7 for Windows from GraphPad Software (La Jolla, CA, USA). Results were considered statistically significant with a *p-*value ≤0.05. Comparisons between groups were performed using one-way ANOVA with multiple comparison. In some cases, direct comparisons between genotypes were made using unpaired Student's *t-*tests. ^*^p ≤ 0.05, ^**^p ≤ 0.01, ^***^p ≤ 0.001, ^****^p ≤ 0.0001.

## Results

### Nox2 Regulates Platelet Activation

**Figure 1:**
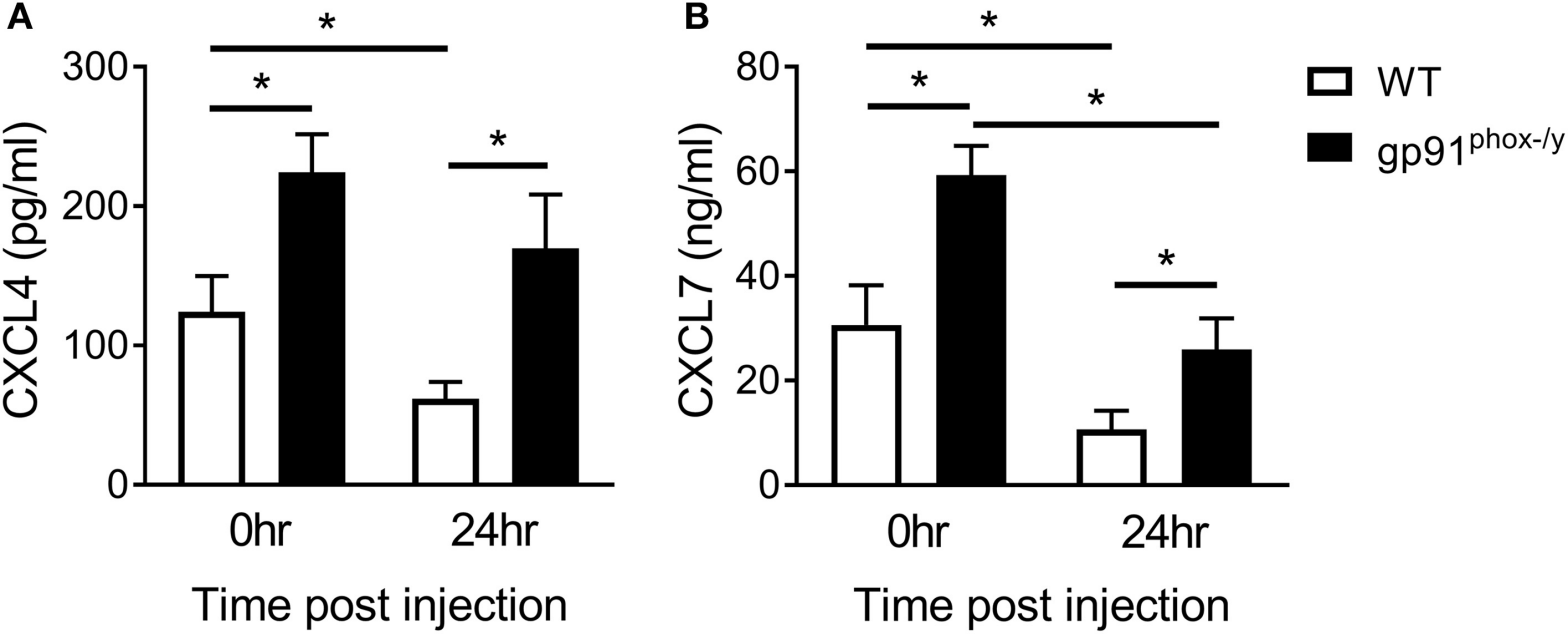
Nox2 regulates platelet activation. Platelet-derived chemokines **(A)** CXCL4 and **(B)** CXCL7 are elevated in the BAL fluid of gp91^phox−/y^ mice at baseline and 24 h after induction of SIRS by peritoneal injection of zymosan, *n* ≥ 8 for CXCL4, *n* ≥ 5 for CXCL7, ^*^p < 0.05.

In view of the findings of hemorrhagic BAL fluid and extensive thrombosis in the lungs of gp91^phox−/y^ mice, we sought evidence of platelet activation in our model. We measured two platelet-derived chemokines, CXCL4 and CXCL7. We found an increase in both chemokines in the BAL fluid of gp91^phox−/y^ mice at baseline and after induction of SIRS by intraperitoneal zymosan injection (Figure 1). Although these neutrophil chemoattractants are increased at baseline, there were minimal/no neutrophils in the BAL fluid of gp91^phox−/y^ mice under unstimulated conditions (7). These data suggest that the presence of CXCL4 and CXCL7 alone is not sufficient to stimulate tissue damage or neutrophil infiltration in the lung. There is a significant decrease in CXCL4 and CXCL7 in the BAL fluid of WT mice 24 h after SIRS induction. CXCL7 but not CXCL4 is reduced in the BAL fluid of gp91^phox−/y^ mice 24 h after SIRS induction (Figure 1).

### Decreased Interaction Between Neutrophils and Platelets in the Peripheral Blood of Mice Lacking Nox2

**Figure 2:**
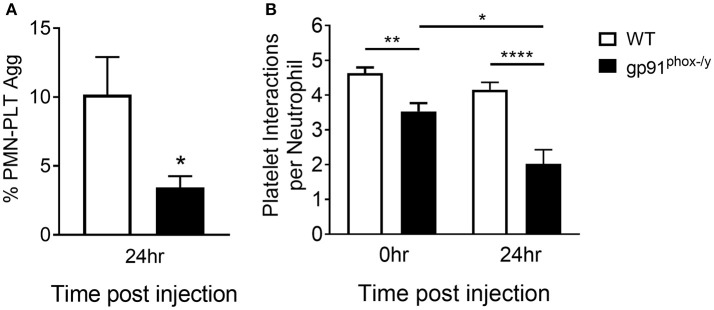
Decreased interaction between neutrophils and platelets in the peripheral blood of mice lacking Nox2. **(A)** A greater percentage of circulating neutrophils in WT mice display platelet markers as demonstrated by dual positivity for CD41 and CD45 by flow cytometry 24 h following induction of SIRS by peritoneal injection of zymosan, *n* = 8, ^*^p < 0.05. **(B)** Using a microfluidic adhesion assay, there are more platelets per neutrophil in the peripheral circulation of WT mice both at baseline and after induction of SIRS, and the interaction is unchanged in WT mice. In contrast, neutrophils from gp91^phox−/y^ mice demonstrate fewer neutrophil-platelet interactions at baseline and the number of aggregates decreases further after SIRS induction, *n* ≥ 47 cells per condition, ^*^p < 0.05, ^**^p < 0.01, ^****^p < 0.0001.

Based on the suggestion that platelets are activated under resting conditions in the Nox2-deficient state, we assessed interactions between neutrophils and platelets in the peripheral blood post-SIRS using two complementary approaches: flow cytometry and a microfluidic adhesion assay. We observed greater frequency of neutrophil-platelet aggregates in the peripheral blood of WT mice at baseline and at 24 h after the induction of SIRS consistent with sequestration of activated platelets in the lung of gp91^phox−/y^ mice. In addition, neutrophil-platelet aggregates were further decreased in the peripheral blood of gp91^phox−/y^ mice post-SIRS while remaining steady in WT mice (Figure 2). Previous results from our lab demonstrate a reduction in the platelet count of gp91^phox−/y^ mice following SIRS (7) consistent with sequestration in the lung and decreased interaction with neutrophils in the peripheral blood of mice lacking Nox2.

### Neutrophil Infiltration in the Alveolar Space of Mice Lacking Nox2

**Figure 3:**
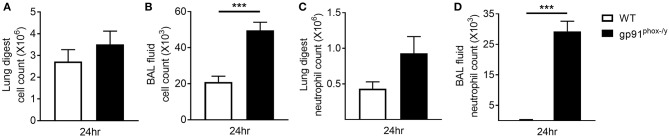
Neutrophilic infiltration in the alveolar space of gp91^phox−/y^ mice following SIRS induction. Total cell count from the lung digest **(A)** and BAL fluid **(B)** of WT (*n* = 8) and gp91^phox−/y^ (*n* = 12) mice 24 h after SIRS induction. Total neutrophil count from the lung digest **(C)** and BAL fluid **(D)** of WT (*n* ≥ 3) and gp91^phox−/y^ (*n* ≥ 5) mice 24 h after SIRS induction, ^***^p < 0.001.

Based on previous studies in our lab (5) demonstrating significant immune cell infiltration in the lung of gp91^phox−/y^ mice, we sought to determine if this infiltration was primarily in the lung interstitium or the alveolar space. In the previous study, lung sections were prepared from lungs without prior BAL. For this study, lungs were harvested after BAL and circulatory perfusion to remove cells from the alveolar space and the vasculature. We counted the total number of cells isolated from the lung digest 24 h post-SIRS and found no difference in total cell number or total neutrophil number between the two genotypes, although there was a trend toward increased neutrophils in the gp91^phox−/y^ mice (Figures 3A,C). In contrast, the total cell number and the total neutrophil number were significantly greater in the BAL fluid of gp91^phox−/y^ mice 24 h following SIRS induction (Figures 3B,D) demonstrating neutrophilic infiltration in the alveolar space.

### Neutrophil ICAM-1 Expression Increases as a Result of the Systemic Inflammatory Response

**Figure 4:**
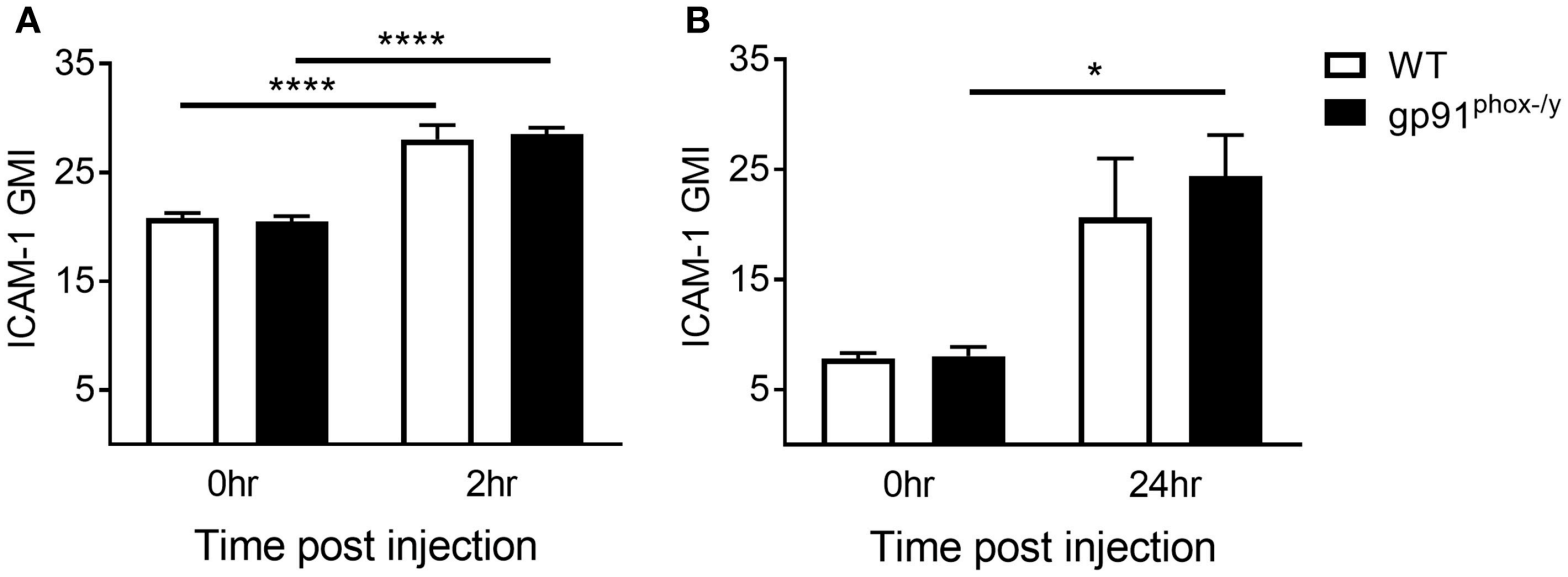
Increase in neutrophil ICAM-1 expression following systemic inflammation. **(A)** Peripheral blood neutrophil ICAM-1 expression at baseline and 2 h following SIRS induction, *n* ≥ 4. **(B)** ICAM-1 expression on neutrophils isolated from the lung at baseline and 24 h following SIRS induction, *n* ≥ 3, ^*^p < 0.05, ^****^p < 0.0001.

We next sought to better understand the mechanisms driving migration of neutrophils to the alveolar space. Leukocyte-endothelial interactions mediated by leukocyte integrins and endothelial ICAM-1 have been well-described (26). Neutrophils are marginated in the lung of both WT and gp91^phox−/y^ mice within 2 h of SIRS induction, and the neutrophils from gp91^phox−/y^ mice express higher levels of CD11b (7). Although neutrophils are marginated in the lung of both WT and gp91^phox−/y^ mice, there is a rapid and significant infiltration of neutrophils into the alveolar space of gp91^phox−/y^ mice only, within 2 h post zymosan injection, and these cells express higher levels of CD11b (7). In contrast to endothelial ICAM-1, relatively little is known about ICAM-1 expression in neutrophils. ICAM-1 upregulation has been demonstrated in response to lipopolysaccharide both *in vitro* and *in vivo* in murine neutrophils (27) and *in vitro* in human neutrophils (28). In the current study, we measured a significant increase in ICAM-1 expression on neutrophils in the peripheral blood of both WT and gp91^phox−/y^ mice 2 h following SIRS induction (Figure 4A). After 24 h of systemic inflammation, neutrophils isolated from the lung of gp91^phox−/y^ mice expressed more ICAM-1 than at baseline (Figure 4B). ICAM-1 expression on neutrophils from the blood or the lung was not different between the two genotypes at the time points tested suggesting that ICAM-1 expression does not account for the difference in neutrophil migration.

### Neutrophils Expressing High Levels of PSGL-1 Migrate to the Alveolar Space in the Absence of Nox2

**Figure 5:**
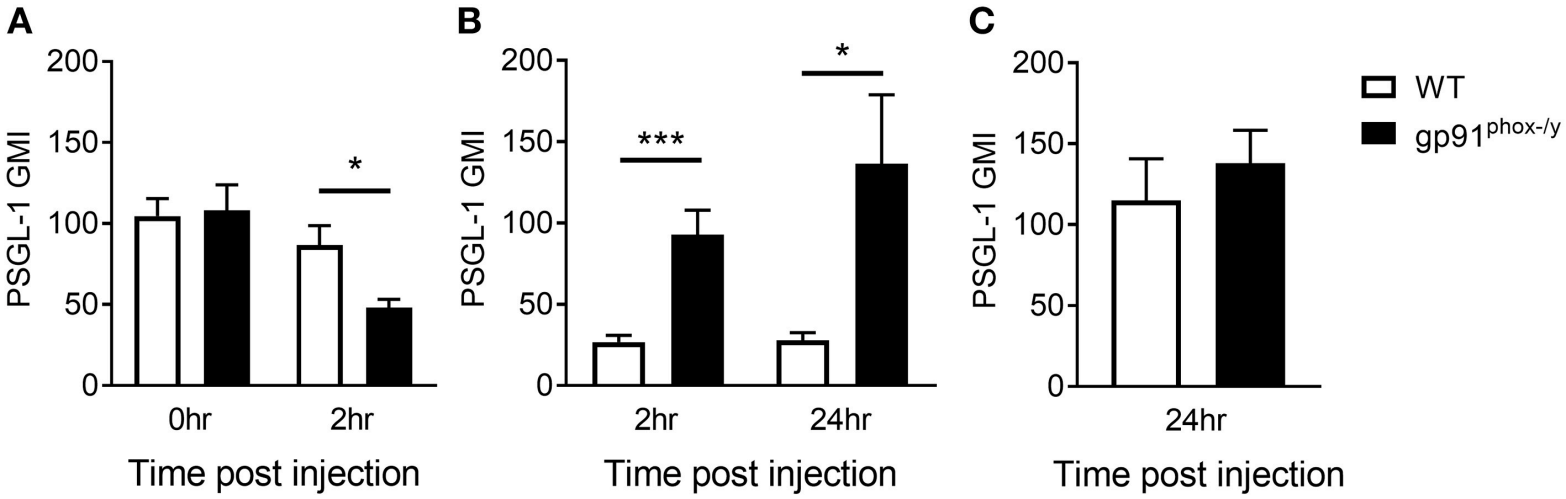
Nox2-deficient neutrophils expressing high levels of PSGL-1 migrate to the alveolar space. **(A)** Peripheral blood neutrophil PSGL-1 expression at baseline and 2 h post zymosan injection, *n* ≥ 3. **(B)** Neutrophils from the BAL fluid of gp91^phox−/y^ mice express more PSGL-1 following SIRS induction than neutrophils from WT mice, *n* ≥ 7. **(C)** PSGL-1 expression on neutrophils isolated from the lung digest 24 h after SIRS induction, *n* ≥ 4, ^*^p < 0.05, ^***^p < 0.001.

Based on the difference in neutrophil-platelet interactions in the peripheral blood, we next sought to measure the neutrophil surface expression of PSGL-1, a receptor of P-selectin on the platelet surface, analyzing circulating PMN, PMN that marginated in the lung, and those that transmigrated to the alveolar space. Although there is no difference in PSGL-1 expression on neutrophils in the peripheral blood of WT and gp91^phox−/y^ mice under resting conditions, there is a rapid decrease in PSGL-1 expression on circulating neutrophils in the gp91^phox−/y^ mice by 2 h post SIRS, with a concomitant increase in PSGL-1 expression on neutrophils in the BAL fluid, suggesting that the “higher” expressing neutrophils have migrated into the alveolar space (Figures 5A,B). At 24 h post SIRS induction, neutrophils in the BAL fluid of gp91^phox−/y^ mice express more PSGL-1 (Figure 5B). There is no difference in PSGL-1 expression on neutrophils isolated from the lung at 24 h (Figure 5C). PSGL-1 on neutrophils binds to P-selectin on activated endothelial cells and platelets resulting in adhesion and migration of cells into inflamed tissues. Considered in combination, these data suggest an environment in the alveolar space of gp91^phox−/y^ mice with highly activated platelets and neutrophils creating optimal conditions for NET formation and tissue damage.

### Nox2 Regulates NET Formation in the Lung

**Figure 6:**
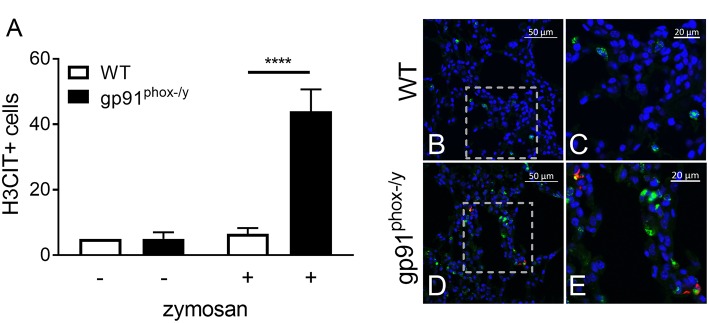
Nox2 represses NET formation in the lung following systemic inflammation. **(A)** Enhanced NET formation is evident as measured by the number of H3CIT positive neutrophils in the lungs of the gp91^phox(−/y)^ mice 24 h after SIRS induction by intraperitoneal injection of zymosan as compared with WT mice or saline injection, *N* = 2 saline injected, and 5–7 zymosan injected, ^****^p < 0.0001. **(B–E)** Representative lung sections demonstrating dual staining for myeloperoxidase (green) and H3CIT (red) in WT **(B–C)** and gp91^phox(−/y)^ **(D–E)** mice 24 h after SIRS induction. DAPI was used to identify the nucleus.

**Figure 7:**
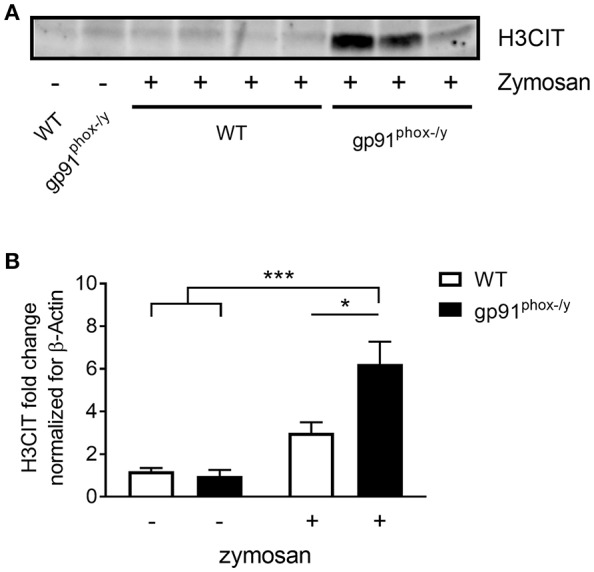
Increased citrullinated H3 in the lung of gp91^phox−/y^ mice following SIRS induction. **(A)** Representative immunoblot showing H3CIT protein from the lung of WT and gp91^phox−/y^ mice 24 h after saline injection or SIRS induction by intraperitoneal zymosan. **(B)** Quantitation of H3CIT in the lung showing an increase in expression in the lung of gp91^phox−/y^ mice, *n* = 5 saline injected and 7 zymosan injected, ^*^p < 0.05, ^***^p < 0.001.

Given the severe lung pathology, neutrophil-platelet crosstalk, and the presence of CXCL4 and CXCL7, chemokines implicated in NET formation, in the alveolar space of gp91^phox−/y^ mice, we investigated lung sections for evidence of NETs. NET formation is characterized by decondensation of chromatin followed by protrusion of nuclear contents outside the cell. Primary granule contents including elastase and myeloperoxidase are commonly found associated with the DNA web (18). NET formation is a regulated inflammatory process resulting in tissue damage and blood vessel occlusion in extreme cases (18, 20, 29, 30). We observed a striking increase in the number of myeloperoxidase and H3CIT dual positive cells in the lung of gp91^phox−/y^ mice 24 h after SIRS induction (Figure 6). H3CIT positive cells were seen in both the interstitium and blood vessels. In addition, H3CIT was increased in whole lung lysate of gp91^phox−/y^ mice 24 h after SIRS induction as compared with saline injected mice and WT mice that were injected with zymosan. Although there is a trend toward increased H3CIT in the lung lysate of WT mice 24 h after SIRS induction, it is not significantly different than H3CIT in the lung lysate of saline injected mice (Figure 7). The increase in NET formation and H3CIT in the lung of gp91^phox−/y^ mice is not due to an overall increase in neutrophils. Although there is a trend toward increased neutrophils in the lung of gp91^phox−/y^ mice 24 h following SIRS induction, it does not reach the level of significance (Figure 3C). Based on the increase in H3CIT in the lung of gp91^phox−/y^ mice, as well as numerous studies demonstrating a requirement for PAD4 in the citrullination of H3 (31), we studied the activity of PAD4 in our model system.

### Nox2 Regulates PAD4 Activity in the Lung

**Figure 8:**
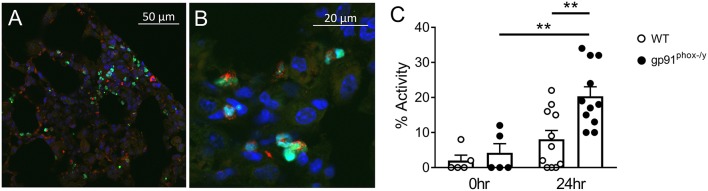
Nox2 represses PAD4 activity in the lung following systemic inflammation. **(A,B)** Lung sections from a gp91^phox−/y^ mouse 24 h following injection of intraperitoneal zymosan demonstrate nuclear localization of PAD4 (green), co-localizing with DAPI (blue) and granular localization of myeloperoxidase (red) staining. **(C)** PAD4 activity in the supernatant recovered from whole lung digest of mice at baseline (*n* = 5) and 24 h (*n* = 11) following injection of zymosan, ^**^p < 0.05.

PAD4 activity has been mechanistically implicated in NET formation in response to multiple stimuli including ROS-dependent and ROS-independent mechanisms (20, 30–32). In our mouse model of systemic inflammation, PAD4 was observed in a nuclear distribution in neutrophils present in the lung of gp91^phox−/y^ mice 24 h following SIRS induction, as expected (Figures 8A,B). Consistent with greater citrullination of H3 in the lung of gp91^phox−/y^ mice following induction of SIRS, PAD4 activity was greater in the lung of mice lacking Nox2 following SIRS induction, and PAD4 activity in the lung of mice lacking Nox2 was significantly higher at 24 h than at baseline (Figure 8C). There was no difference between the genotypes in PAD4 activity at baseline.

## Discussion

Advances in modern intensive care technology have decreased mortality during the acute inflammatory phase of numerous illnesses. Although most patients now survive the initial insult, a significant subset develop life-threatening inflammatory multi-organ failure, and there are no directed therapies to treat these organ-specific dysfunctions. A better understanding of the underlying mechanisms that govern organ-specific inflammation will help define targeted therapies for this vulnerable population of patients. In previously published work, we demonstrated a critical protective role for the NADPH oxidase 2 in the development of systemic inflammation and multi-organ injury in the setting of sterile SIRS (5, 6). In the current study, we sought to unravel the specific mechanisms by which Nox2 might specifically protect the lung, and to characterize the events that result in neutrophilic infiltration in the alveolar space, resulting in severe thrombosis, hemorrhage, and mortality.

The lung has been recognized as a unique organ in terms of the signaling mechanisms governing neutrophil migration and activation (33–35). We recently demonstrated that Nox2 in alveolar macrophages plays a homeostatic role in repressing basal chemokine secretion into the alveolar space (7). Here, we significantly expand current understanding of the protective role of Nox2 in lung homeostasis with three novel findings. First, we demonstrate that Nox2-derived ROS participate in the repression of platelet activation in the lung under resting and stimulated conditions. This role likely alters neutrophil-platelet interactions and has downstream impact on neutrophil-mediated tissue damage. Second, neutrophilic inflammatory injury to the lung is characterized by widespread alveolar NET formation in the absence of Nox2. This display of NET formation in the Nox2-deficient state is critically important to the way we think about oxidants. Finally, we demonstrate enhanced PAD4 activity in the lung of Nox2 deficient mice under conditions that promote NET generation.

The initial report of NETs, and what was initially termed NETosis, described an extrusion of nuclear contents decorated with primary granule proteases (17). The early studies of NETs characterized NETosis as a unique mechanism of cell death with a role in bacterial killing and a requirement for Nox2-derived ROS (36). Numerous laboratories extended these findings for Nox2-dependent NETs, although in many cases the phorbyl ester, PMA, was utilized as the primary stimulus for NET generation. There is a growing list of stimuli that induce NET formation, and a broad range of intracellular signaling pathways involved in these processes. Moreover, it is now recognized that NETs have a role extending beyond host defense, with NET mediated tissue damage playing a role in chronic inflammatory diseases and a growing understanding of involvement in autoimmunity (18, 37). Relevant to our work, it is clear that not all NET formation is Nox2-dependent, and there may, in fact, be completely unique signaling pathways governing these distinctive forms of NET generation (20, 30, 32, 38). To our knowledge, our report provides the first description of Nox2-mediated repression of NET formation under inflammatory conditions.

Whereas, much of the published literature surrounding NET formation utilizes reductionist *in vitro* analyses, we sought to understand the mechanisms of lung injury in an animal model. In our *in vivo* model of systemic inflammation, there are likely diverse and interdependent cell signaling pathways involved. The importance of integrin-mediated signaling via CD11b has been demonstrated in sterile inflammation (39). Our studies have demonstrated upregulation of CD11b on the neutrophil surface in the absence of Nox2 function *in vitro* and *in vivo* (7, 8), specifically in neutrophils reaching the alveolar space in mice. In the current study, we extend our observations, by focusing on neutrophil-platelet interactions. The finding of enhanced levels of platelet chemokines in the alveolar space of Nox2-deficient mice under unstimulated conditions suggests that Nox2 has an active role in repressing platelet activation. Importantly, platelet-derived CXCL4 has been demonstrated to be critically involved in the pathogenesis of acute lung injury from both viral and bacterial etiologies (40–42). CXCL4 is involved in platelet aggregation and plays a role in coagulation. CXCL7 exists in monomer, dimer, and tetramer form. The monomer form is a potent neutrophil agonist, and the dimer form has been implicated in neutrophil-platelet crosstalk (43). The expression of these platelet chemokines, and also alveolar macrophage derived chemokines (7) is not sufficient, in our model system, to elicit neutrophil migration under resting conditions. However, once systemic inflammation is initiated, additional changes in the neutrophil activation state become apparent allowing the progression of lung injury. PSGL-1 is an adhesion molecule expressed on neutrophils that binds to P-selectin expressed on activated endothelial cells and platelets. This binding of PSGL-1 to P-selectin facilitates neutrophil adhesion and has been implicated in NET formation (14–16, 30, 44). In our model, while there are no differences in PSGL-1 expression on neutrophils in healthy mice, there is significant upregulation on neutrophils that have migrated to the alveolar space of gp91^phox−/y^ mice. We postulate that the presence of macrophage-derived cytokines (7), activated platelets, NET-inducing chemokines, and the upregulation of adhesion molecules combine to create an environment favorable to NET formation.

The notion that platelets are directly involved in NET formation is not novel (14). In murine models of both ventilator-induced lung injury and transfusion-related lung injury activated platelets and chemokine release are implicated in the development of NETs (15, 16, 29, 39). Human *in vitro* studies also support direct involvement of platelets in the activation of NET formation (45). Microparticles from activated platelets express high-mobility group box 1 protein, a damage-associated molecular pattern, and promote vasculopathy and NET formation in patients with systemic sclerosis and in a murine model (46). Moreover, platelet-mediated NET formation has already been demonstrated to be Nox2-independent (47). The specific signaling pathways allowing platelet activation to promote NET formation have not been clearly elucidated and likely involve the interplay of numerous interrelated pathways. Our *in vivo* model of SIRS advances our understanding of NET formation in the setting of systemic inflammation and provides evidence for Nox2-mediated repression of NET formation.

Although there are vast arrays of signaling intermediates implicated upstream of NET formation induced by different stimuli, a growing literature supports a critical role for PAD4 as a key mediator of chromatin decondensation. PAD4 is one of five known PADs and the only one with a nuclear localization sequence, and is expressed primarily in granulocytes, whereas the other PADs have broader expression. PADs catalyze a post-translational modification resulting in the de-imination or citrullination of arginine (48). Histone citrullination appears to be the essential step directly upstream of the extrusion of nuclear contents, but the signals leading to PAD4 activation are not fully understood. Using murine knockout models, neutrophils lacking PAD4 cannot generate NETs *in vitro* (49) or *in vivo* (50), and display increased susceptibility to certain bacterial infections (48). As further demonstration of the physiologic relevance of these observations, a PAD4 inhibitor, Cl-amidine, provided protection in a lethal model of polymicrobial sepsis in mice (51). *In vitro*, a highly selective PAD4 inhibitor, GSK 484, inhibits citrullination in human and murine neutrophils and blocks NET formation in response to ionomycin and bacterial stimuli (31). These recent studies have attracted greater attention based on the potential for these inhibitors to be used therapeutically in inflammatory disease. There are several lines of evidence suggesting that dysregulation of PAD4 activity plays a role in the pathogenesis of rheumatoid arthritis in both murine models of arthritis and human neutrophil studies. Moreover, several single nucleotide polymorphisms in PAD4 have been associated with susceptibility to rheumatoid arthritis (52). Our data suggest that off target inhibition of Nox2-derived ROS by broad spectrum anti-oxidant therapies might increase PAD4 activity thus enhancing inflammation.

In summary, the current study significantly advances our understanding of the critical role of Nox2-derived ROS in the maintenance of immune homeostasis in the lung and in the resolution of inflammation following a systemic inflammatory insult. The lung is a vital organ where immune cells come in very close contact with one another and the external environment. There is the potential for the introduction of inflammatory stimuli on a routine basis. In this study, we demonstrate a role for Nox2 in the repression of basal platelet chemokine secretion, in the suppression of neutrophil adhesion molecule upregulation, and in the regulation of a key neutrophil enzyme known to participate in the formation of NETs. The absence of Nox2 alone is insufficient to induce tissue injury. However, the absence of Nox2 creates an environment of low level inflammation that predisposes vulnerable organs to severe injury in the setting of systemic inflammation. In order to improve outcomes for patients who experience significant inflammatory organ injury, it is critical to consider the multifaceted role of ROS in the inflammatory response.

## Data Availability

All datasets generated for this study are included in the manuscript and/or the supplementary files.

## Ethics Statement

All studies were approved and conducted under the oversight of the Institutional Animal Care and Use Committee at the University of Texas Southwestern Medical Center.

## Author Contributions

JH, MC, RP, and NA planned and conducted experiments. MC performed all microscopy and image analysis. JH, RP, NA, DS, and JM analyzed data and contributed to the study design. JH, MC, and NA prepared figures for the manuscript. JH and JM wrote the manuscript.

### Conflict of Interest Statement

The authors declare that the research was conducted in the absence of any commercial or financial relationships that could be construed as a potential conflict of interest.

## Abbreviations

BAL: Bronchoalveolar Lavage
H3CIT: Citrullinated Histone H3
CXCL: CXC Chemokine Ligand
Nox2: NADPH Oxidase 2
NET: Neutrophil Extracellular Trap
PAD: PeptidylArginine Deiminase
PSGL-1: P-selectin Glycoprotein 1
ROS: Reactive Oxygen Species
SIRS: Systemic Inflammatory Response Syndrome.
